# Coverage Dependent Variation of the Adsorption Structure of 2-Thiophenecarboxaldehyde on the Ge(100)-2 × 1 Reconstructed Surface

**DOI:** 10.3390/molecules180910301

**Published:** 2013-08-26

**Authors:** Myungjin Lee, Minjeong Shin, Hangil Lee

**Affiliations:** Department of Chemistry, Sookmyung Women’s University, Seoul 140-742, Korea; E-Mails: mopoyo@sookmyung.ac.kr (M.L.); smj5757@sookmyung.ac.kr (M.S.)

**Keywords:** HRPES, DFT calculation, 2-thiophenecarboxaldehyde, Ge(100)-2 × 1 reconstructed surface, adsorption structure

## Abstract

High-resolution photoemission spectroscopy (HRPES) measurements were collected and density functional theory (DFT) calculations were performed to track the exposure-dependent variation of the adsorption structure of 2-thiophenecarboxaldehyde (C_4_H_3_SCHO: TPCA) on the Ge(100) 2 × 1 reconstructed surface at room temperature. In an effort to identify the most probable adsorption structures on the Ge(100)-2 × 1 reconstructed surface, we deposited TPCA molecules at low exposure and at high exposure and compared the differences between the electronic features measured using HRPES. The HRPES data suggested three possible adsorption structures of TPCA on the Ge(100)-2 × 1 reconstructed surface, and DFT calculations were used to determine the plausibility of these structures. HRPES analysis corroborated by DFT calculations, indicated that an S-dative bonded structure is the most probable adsorption structure at relatively low exposure levels, the [4 + 2] cycloadduct structure is the second most probable structure, and the [2 + 2]-C=O cycloadduct structure is the least probable structure on the Ge(100)-2 × 1 reconstructed surface at relatively high exposure levels.

## 1. Introduction

Recently, many research groups have been studying structures of biomolecules adsorbed onto semiconductor surfaces in connection with the development of bio-related applications [1,2,3,4,5]. The Ge(100)-2 × 1 reconstructed surface provides an excellent model of a semiconductor surface because the Ge(100)-2 × 1 reconstructed surface displays zwitterionic character [6,7]. The zwitterionic dimers of the Ge(100)-2 × 1 reconstructed surface act as Lewis bases and acids, which facilitate the adsorption of molecules with multifunctional groups [8,9,10].

Recent studies have shown that thiol groups react with Ge(100)-2 × 1 reconstructed surface under photo-excitation with 510 nm light. This reaction is useful as a photo-induced thiol detector [11,12]. In terms of developing the other thiol detection applications, we have examined sulfur-containing compounds to understand how these molecules behave on the Ge(100)-2 × 1 reconstructed surface at room temperature. 2-Thiophenecarboxaldehyde (TPCA) is a good test molecule as a detector because its molecular structure is relatively simple and sensitive.

**Figure 1:**
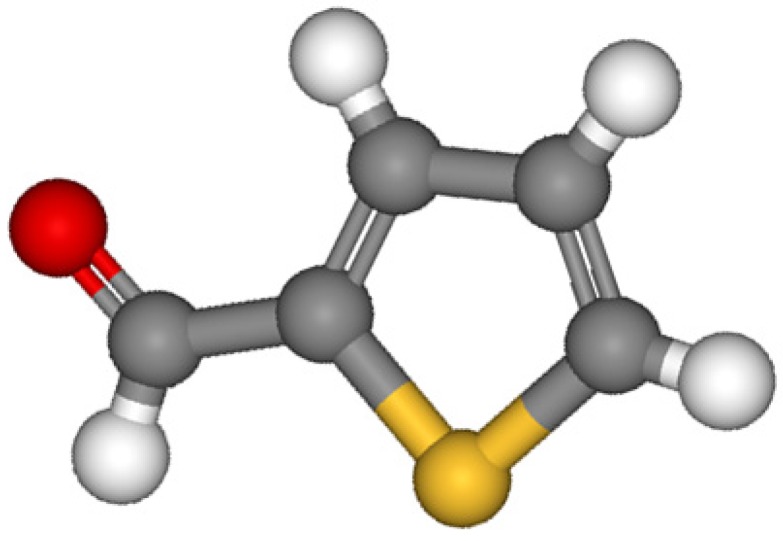
The structure of the TPCA molecule, which has a sulfur (yellow), an oxygen (red), five carbons (gray), and four hydrogens (white), respectively.

Figure 1 shows the structure of a TPCA molecule containing a sulfur atom in an aromatic ring and an aldehyde functional group. Since Kurt Alder and Otto Diels first discovered the Diels–Alder reaction and were awarded the Nobel Prize in Chemistry in 1950, this reaction has been actively studied in a variety of chemical fields [13,14,15,16,17,18]. Contrary to what is known about the Diels-Alder reaction which occurs in aqueous state, TPCA is expected to form several cycloaddition structures under UHV conditions through reactions involving the TPCA diene and the dimer of the Ge(100)-2 × 1 reconstructed surface atoms.

Previously, we reported that thiophene could adsorb onto the Ge(100)-2 × 1 reconstructed surface. Hence, we expected that similar adsorption properties are possible for the TPCA molecule [19]. Thiophene adsorption proceeded via a Lewis acid-base reaction to form two distinct configurations on the Ge(100)-2 × 1 reconstructed surface. At a low exposure (0.25 mL), thiophene formed one-dimensional (1-D) molecular chain structures in an S-dative bonded structure. Beyond exposure of 0.25 mL, the stable [4 + 2] cycloaddition adducts were observed [20]. Here, we sought to compare the adsorption structures of thiophene and TPCA on the Ge(100)-2 × 1 reconstructed surface using high-resolution photoemission spectroscopy (HRPES) and density functional theory (DFT) calculations.

## 2. Results and Discussion

**Figure 2:**
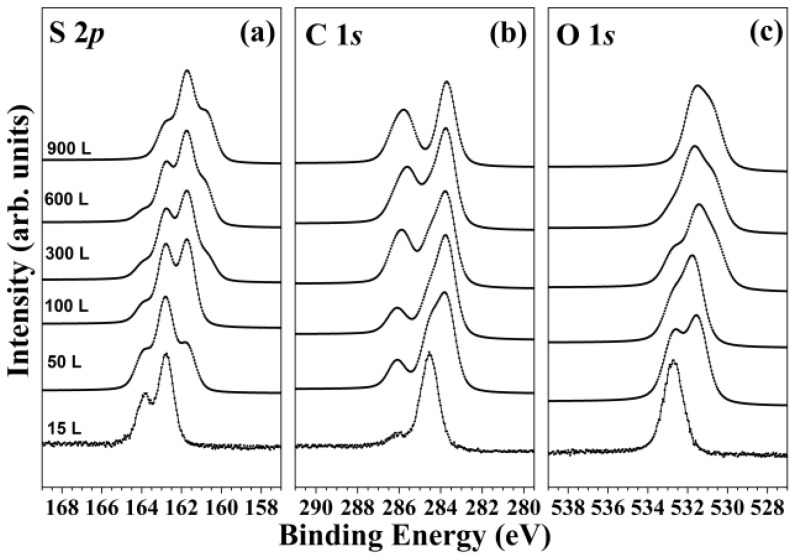
(**a**) S 2*p*, (**b**) C 1*s*, and (**c**) O 1*s* core-level spectra of TPCA adsorbed onto the Ge(100) surface over a range of surface exposure levels.

The TPCA molecule core-level spectra were obtained to examine the sulfur, carbon, and oxygen states. Figure 2 shows the exposure-dependent variations of the TPCA bond characteristics after adsorption onto the Ge(100)-2 × 1 reconstructed surface at room temperature. The exposure-dependent electronic and adsorption structures were obtained from the HRPES data. Figure 2(a) shows the S 2*p* core-level spectra of 15 L to 900 L TPCA adsorbed onto the clean Ge(100)-2 × 1 reconstructed surface at room temperature. The S 2*p* core-level spectra showed a doublet structure due to the presence of the S 2*p*_3/2_ and S 2*p*_1/2_ peaks, and all spectra could be fit using a 2:1 peak area ratio, a 1.1 eV spin-orbit splitting coefficient [19]. The S 2*p* peak positions were found to vary with the TPCA exposure. A sample comprising 15 L TPCA adsorbed onto the Ge(100)-2 × 1 reconstructed surface (initial exposure) yielded a single feature at 162.8 eV (the S 2*p*_3/2_ position). An additional distinct peak (a small peak on the right-hand shoulder around 162.0 eV) was observed at an exposure level of 100 L TPCA, suggesting that another possible TPCA adsorption structure was present on the Ge(100)-2 × 1 reconstructed surface. At 600 L, the distinct shape of a sulfur peak at lower binding energies was observed at 161.0 eV. This peak became more prominent at 900 L TPCA. Figure 2(a) shows that the TPCA molecules adsorbed in any of three distinct possible structures on the Ge(100)-2 × 1 reconstructed surface. Curve fits were used to quantify the ratios of these adsorption structures, discussed further below.

The C 1*s* and O 1*s* core-level spectra were also obtained and analyzed in the same way as the S 2*p* core-level spectra. Figure 1b and Figure 1c showed similar trends to those observed in Figure 2(a). The electronic structure was found to depend on the exposure.

**Figure 3:**
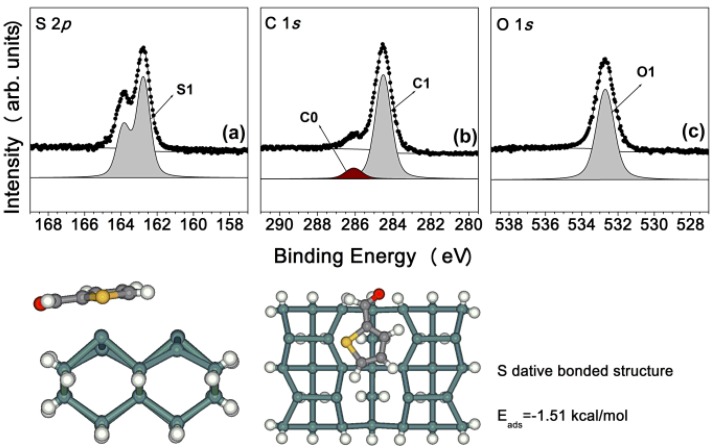
(**a**) S 2*p*, (**b**) C 1*s*, and (**c**) O 1*s* core-level spectra for the sample comprising 15 L TPCA adsorbed onto a clean Ge(100)-2 × 1 reconstructed surface at room temperature. The dots indicate the experimental values, and the solid lines represent the results of peak fitting. The bottom figure shows the DFT calculation result for an S dative bonded structure.

The analysis for bonding features and electronic properties of the possible structures examined was based on the HRPES spectra. We began with an examination of Figure 3, which presents a series of S 2*p*, C 1*s*, and O 1*s* core-level spectra of the Ge(100)-2 × 1 reconstructed surface exposed to 15 L TPCA at room temperature. The S 2*p* core-level spectrum [at 162.8 eV in Figure 3(a)] indicates the presence of an S atom adsorbed onto the Ge(100)-2 × 1 reconstructed surface with a weak bonding energy. This bond energy was consistent with an S dative bonding structure (S1) identified in the context of thiophene adsorption structures on the Ge(100)-2 × 1 reconstructed surface [19,20]. The C 1*s* core-level spectrum, Figure 3(b), indicates the presence of two different carbon atoms: a high-intensity peak was observed at 284.5 eV (C1) and a lower-intensity peak was observed at 286.1 eV (C0). The theory of the Pauling electronegativity [21,22] and the peak intensities predict that the C1 (-C_4_H_3_S) peak arose from the heterocyclic ring of the TPCA molecule and the C0 peak arose from the CHO- group in the TPCA molecule. These two peaks were expected in the context of the S-dative bonded structure, and no other strong covalent bonding features were observed in the C 1*s* core-level spectrum. Figure 3(c) shows the O 1*s* core-level spectrum obtained from the sample. The peak observed at 532.7 eV (O1) indicated that no oxygen atoms participated in the TPCA adsorption structure at 15 L deposition. This peak was expected to originate from an un-adsorbed aldehyde present as a contaminant in the TPCA sample. At a exposure of 15 L, only an S-dative bonded structure on the Ge(100)-2 × 1 reconstructed surface was expected, based on the S 2*p* core-level spectra. DFT calculations were performed to further test the reasonableness of the S-dative bonded structure model. The bottom of Figure 3 shows a ball model of the adsorption structure and DFT calculations reveals a negative adsorption energy (−1.51 kcal/mol) for this structure, indicating that an S-dative bonded structure is predicted to be stable at room temperature.

**Figure 4:**
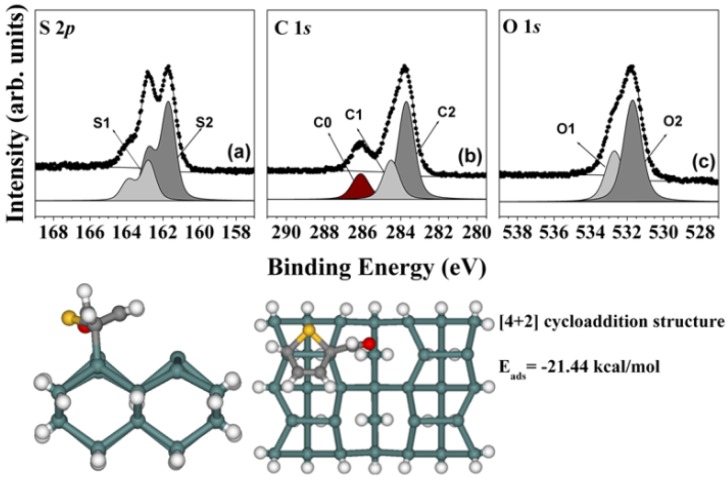
(**a**) S 2*p*, (**b**) C 1*s*, and (**c**) O 1*s* core-level spectra for a sample comprising 100 L TPCA adsorbed onto the clean Ge(100) surface at room temperature. The dots indicate the experimental values and the solid lines represent the results of peak fitting. The bottom figure shows the DFT calculation result for a [4 + 2] cycloadduct structure.

Samples comprising 25 L and 50 L TPCA were prepared. The spectra obtained after deposition of 50 L show any remarkable features; however, the spectra of the sample prepared with 100 L TPCA indicate distinct bonding features at 162.8 eV (S1) and 161.7 eV (S2). By comparison to the thiophene study on the Ge(100)-2 × 1 reconstructed surface [19,20], the S2 peak corresponded to a -C-S-C- type of sulfur, indicating that the sulfur atom was not directly adsorbed onto the Ge(100)-2 × 1 reconstructed surface. Other plausible adsorption structures must be considered. TPCA molecules include two double bonds that can form a [4 + 2] cycloadduct structure. DFT calculation data predicted that this structure would provide stable adsorption energy (bottom of Figure 3); therefore, this structure could be assigned as arising from the [4 + 2] cycloadduct structure. The bonding strength of the [4 + 2] cycloadduct structure was relatively intense, so these two peaks were assigned to the S-dative bonded structure (S1) and the [4 + 2] cycloadduct structure (S2), respectively. We next investigated the carbon features of the TPCA molecule, as shown in Figure 4(b). The additional new peak (C2) observed at 283.7 eV was assigned to a strong covalent bond in the [4 + 2] cycloadduct structure. The C 1*s* core-level spectrum was consistent with a [4 + 2] cycloadduct structure on the Ge(100)-2 × 1 reconstructed surface. Figure 4(c) shows the O 1*s* core-level spectrum obtained at 100 L TPCA. Two remarkable peaks were observed at 532.7 eV (O1) and 531.7 eV (O2). We compared these peak intensities with binding energies, and this shows that these peaks indicate the existence of [4 + 2] cycloadduct structure [19,20]. Thus, all three types of peak were identified as arising from the [4 + 2] cycloadduct structure. The DFT calculations further supported that the [4 + 2] cycloadduct structure is a possible adsorption structure on the Ge(100)-2 × 1 reconstructed surface at room temperature. The bottom panel of Figure 4 shows the stable adsorption structure of a [4 + 2] cycloadduct product with an adsorption energy of E_ads_ = −21.44 kcal/mol. The DFT calculations predicted that the [4 + 2] cycloadduct bonded structure would be more stable than the S-dative bonded structure at this exposure.

**Figure 5:**
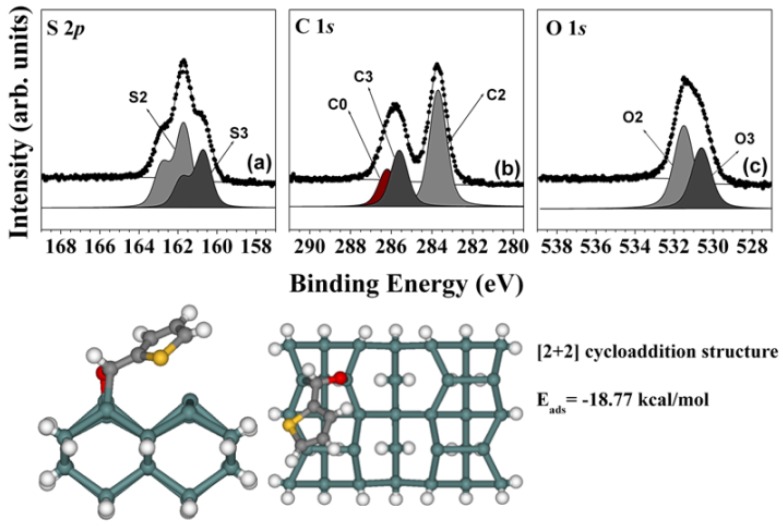
(**a**) S 2*p*, (**b**) C 1*s*, and (**c**) O 1*s* core-level spectra of a sample comprising 900 L TPCA adsorbed onto a clean Ge(100)-2 × 1 reconstructed surface at room temperature. The dots indicate experimental values and the solid lines represent the results of peak fitting. The bottom figure shows the DFT calculation results and predicted structure of the [2 + 2]-C=O cycloadduct product.

Finally, as the TPCA exposure was increased up to 900 L, new remarkable features were observed, which were not observed in the system of thiophene adsorbed on the Ge(100)-2 × 1 reconstructed surface, as shown in the S 2*p*, C 1*s*, and O 1*s* core-level spectra of Figure 5. Two bonding features at 161.7 eV (S2), and 160.7 eV (S3) were observed in the S 2*p* core-level spectra. The 162.8 eV peak at low TPCA exposure levels [S1; see Figure 3(a)] appeared to have been exchanged into the two peaks in Figure 5(a) (S2 and S3). Based on our previous explanation, the S3 peak could be attributed to -C-S-C=O type of sulfur, which would be consistent with the binding energy of the S3 peak [19,20]. The relationship between two peaks was examined in view of the C 1*s* core-level spectrum, shown in Figure 5(b), which featured three different types of carbon atoms. The new peak located at 285.7 eV (marked as C3) was thought to have arisen from a strong covalent bonded structure. This peak differed from the peak of the [4 + 2] cycloadduct bonded structure and corresponded to another strong covalent bonding feature in the C 1*s* core-level spectra. The O 1*s* core-level peaks in Figure 5(c) show oxygen atom electronic features at 531.5 eV (O2), and 530.6 eV (O3). The new bonding feature (O3) appeared to have arisen from a distinct direct adsorption structure that included an oxygen atom. The bonding configuration could have involved a [2 + 2]-C=O cycloadduct bonded structure. DFT calculations of this [2 + 2]-C=O cycloadduct bonded structure were performed to estimate the likelihood that this structure could be stable at room temperature. Interestingly, as shown in the bottom panel of Figure 5, this adsorption structure was predicted to be stable, with E_ads_ = −18.77 kcal/mol. This structure was not observed in the thiophene on the Ge(100)-2 × 1 reconstructed surface system. These results indicate that TPCA adsorbed onto the Ge(100)-2 × 1 reconstructed surface in any of three adsorption structures, depending on the TPCA exposure level.

**Figure 6:**
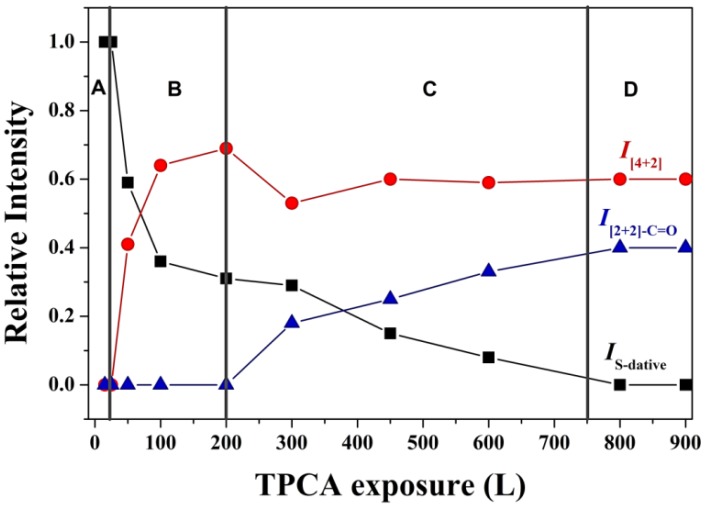
Plot showing the relative HRPES peak intensities for the three TPCA adsorption structures on the Ge(100)-2 × 1 reconstructed surface at room temperature, as a function of exposure. Each black, red, and blue colored mark indicates the variation of relative intensities among the S-dative bonded structure (marked as black colored square), [4 + 2] cycloadduct structure (marked as red colored circle), and [2 + 2]-C=O cycloadduct bonded structure (marked as blue colored triangle) at each exposure, respectively.

The relative intensities of the HRPES peaks are summarized in Figure 6 to show the relative populations of the three possible adsorption structures on the Ge(100)-2 × 1 reconstructed surface, as a function of the exposure level. The adsorption structure populations may be divided into four types (A, B, C, and D). At exposure levels below 25 L TPCA, molecules occupied the A region; at exposure levels between 25 L and 200 L TPCA, molecules occupied the B region; at exposure levels between 200 L and 750 L TPCA, molecules occupied the C region; and at exposure levels beyond 750 L TPCA, molecules occupied the D region. The adsorption structures on the Ge(100)-2 × 1 reconstructed surface clearly varied with the exposure level. The S-dative bonded structure (black) was preferred in region A, and its prevalence decreased rapidly over region B to region D. This result indicated that most of the S-dative bonded structures were formed during the initial deposition, up to an exposure of 200 L. Region B included two distinct types of adsorption structures: the S-dative bonded structure and the [4 + 2] cycloadduct bonded structure. The red plot shows that the [4 + 2] cycloadduct structure became prevalent in region B, and this prevalence maintained up to exposure of 900 L. We infer that the [4 + 2] cycloadduct structure was the most stable structure, as its prevalence was maintained at all exposure levels. Finally, the [2 + 2]-C=O cycloadduct structure (the blue plot) slowly gained in prevalence in region C. The [2 + 2]-C=O cycloadduct structure was less prevalent than the [4 + 2] cycloadduct structure; however, the concentration increased monotonically. These intensity trends indicate that the S-dative bonded structure, which was prevalent at low exposure levels, was converted to the [2 + 2]-C=O cycloadduct structure around 350 L exposure (region C). The intensity ratio data as a function of the TPCA exposure on the Ge(100)-2 × 1 reconstructed surface in reported in Table 1.

**Table 1:** Relative intensities of the three TPCA adsorption structures on the Ge(100)-2 × 1 reconstructed surface at room temperature, as a function of TPCA exposure. I_S-dative_, I_[4 + 2]_, and I_[2 + 2]-C=O_ indicate the relative intensity at each exposure.

TPCA exposure (L)	I_S-dative_	I_[4 + 2]_	I_[2 + 2]-C=O_
15	1.00	0.00	0.00
25	1.00	0.00	0.00
50	0.59	0.41	0.00
100	0.36	0.64	0.00
200	0.31	0.69	0.00
300	0.29	0.53	0.18
450	0.15	0.60	0.25
600	0.08	0.59	0.33
800	0.00	0.60	0.40
900	0.00	0.60	0.40

## 3. Experimental

Ge(100)-2 × 1 reconstructed surfaces (*p*-type, R = 0.10–0.39 Ω) were cleaned by several cycles of sputtering with 1 keV Ar + ions at 700 K for 20 minutes, followed by annealing at 900 K for 10 minutes. The cleanliness of the Ge(100)-2 × 1 reconstructed surface was checked using low-energy electron diffraction (LEED). 2-Thiophenecarboxaldehyde (C_4_H_3_SCHO, 99% purity) was purchased from Sigma-Aldrich (Yong-in, Korea) and was further purified through several sublimation and pumping cycles to remove dissolved gases prior to exposure to the Ge(100)-2 × 1 reconstructed surface.

HRPES measurements were collected at the 10D beamline of the Pohang Accelerator Laboratory. The S 2*p*, C 1*s*, and O 1*s* core-level spectra were obtained using a PHOIBOS 150 electron energy analyser equipped with a two-dimensional charge-coupled device (2D CCD) detector (Specs GmbH, Berlin, Germany) using photon energies of 235, 340, and 588 eV to enhance the surface sensitivity. The binding energies of the core-level spectra were calibrated with respect to the clean Au 4*f* core-level spectrum (84.0 eV) collected at the same set of photon energies. The base pressure in the chamber was maintained below 9.5 × 10^−11^ Torr. All spectra were recorded in the normal emission mode. The core-level spectra were carefully analysed using a standard nonlinear least squares fitting procedure with Voigt functions [23].

DFT calculations were used to analyze the energetics of the potential pathways for the tyrosine and phenylalanine reactions on the Ge(100)-2 × 1 reconstructed surface. All DFT calculations of the adsorption energies were performed using the JAGUAR 9.1 software package, which applied a hybrid density functional method and included Becke’s three-parameter nonlocal-exchange functional with the correlation functional of Lee–Yang–Parr (B3LYP) [24,25]. The calculations were performed using a four-dimer (Ge_35_H_32_) cluster model. The geometries of important local minima on the potential energy surface were determined at the B3LYP/LACVP** level of theory. The LACVP** basis set is a mixed basis set that uses the LACVP basis set to describe the Ge atoms and the 6-31G basis set to describe the remaining atoms. The LACVP basis set is useful for describing atoms heavier than Ar in the periodic table and is based on the Los Alamos effective core potentials developed by Hay and Wadt [26,27]. Optimization of each cluster was performed by fixing the bottom two layers of the Ge atoms in the ideal Ge crystal positions, permitting the top layer of Ge atoms (including the dimer atoms) and the atoms of the chemisorbed adsorbate to relax. The geometries of important local minima and transition states on each energy diagram were calculated. The local minima and transition states in the optimized structures were verified using the same set of basis sets [28].

## 4. Conclusions

We investigated the adsorption geometries and the exposure-dependent bonding states of TPCA molecules adsorbed onto the Ge(100)-2 × 1 reconstructed surface using HRPES and DFT calculations. The HRPES data suggested that the TPCA molecules adsorbed onto the Ge(100)-2 × 1 reconstructed surface in three possible adsorption structures. The S-dative bonded structure was prevalent at low (15 L TPCA) exposure levels. The [4 + 2] cycloadduct structure, which includes stronger bonds, was prevalent around 100 L TPCA. The [2 + 2]-C=O cycloadduct structure was prevalent at 900 L TPCA, and this structure was not observed in previous studies of thiophene adsorbed onto the Ge(100)-2 × 1 reconstructed surface. Oxygen atoms are strongly electronegative and can form a [2 + 2]-C=O cycloadduct structure more easily than the carbon atoms of thiophene can form a [2 + 2]-C=C cycloadduct structure on the Ge(100)-2 × 1 reconstructed surface. The electronegativity of oxygen could explain why oxygen atoms adsorb at the down dimer on the Ge(100) surface, which has a relatively positive charge. Ge(100)-2 × 1 reconstructed surfaces display zwitterionic properties that allow oxygen atoms to adsorb onto Ge(100) down dimers. The energies of the proposed adsorption structures were predicted using DFT calculations. Three negative energy values were obtained for the S-dative bonded structure, the [4 + 2] cycloadduct structure, and the [2 + 2]-C=O cycloadduct structure, and these energies agreed well with the HRPES data. We concluded the [4 + 2] cycloadduct structure was the most stable adsorption structure on the Ge(100)-2 × 1 reconstructed surface at room temperature.
